# Visualizing the Hidden Half: Plant-Microbe Interactions in the Rhizosphere

**DOI:** 10.1128/mSystems.00765-21

**Published:** 2021-09-14

**Authors:** Pubudu P. Handakumbura, Albert Rivas Ubach, Anil K. Battu

**Affiliations:** a Environmental Molecular Sciences Laboratory, Pacific Northwest National Laboratory, Richland, Washington, USA

**Keywords:** 3D visualization of roots, metabolomics, plant-microbe interactions, rhizosphere hot spots, root exudates

## Abstract

Plant roots and the associated rhizosphere constitute a dynamic environment that fosters numerous intra- and interkingdom interactions, including metabolite exchange between plants and soil mediated by root exudates and the rhizosphere microbiome. These interactions affect plant fitness and performance, soil health, and the belowground carbon budget. Exploring and understanding the molecular mechanisms governing ecosystem responses via rhizosphere interactions allow the rational and sustainable design of future ecosystems. However, visualizing the plant root system architecture with spatially resolved root exudate and microbiome profiles along the root in its native state remains an ambitious grand challenge in rhizosphere biology. To address this challenge, we developed a three-dimensional (3D) root cartography platform to accurately visualize molecular and microbial constituents and their interactions in the root-rhizosphere zone.

## COMMENTARY

The rhizosphere, the soil layer tightly associated with a plant root, is one of the most dynamic interfaces on earth, harboring a multitude of intra- and interkingdom interactions. In addition, the root system architecture itself is highly plastic and responds to both biotic and abiotic influences in the rhizosphere. Plant roots exude a staggeringly diverse collection of small molecules, including secondary metabolites that shape the rhizosphere. The complexity and diversity of the root exudates speak to the intricacies of the chemical language that plants utilize to control rhizosphere interactions. Numerous chemical, physical, and biological factors in the rhizosphere can also influence the root exudate profiles (1). Modulation of the root exudate composition is an evolutionary survival strategy that plants use to adapt to or avoid adverse belowground conditions, e.g., by attracting beneficial microbes, releasing toxic compounds to combat pathogens, changing the soil pH, and chelating toxic molecules (2, 3). The rhizosphere microbiome also responds to the developmental stages of its plant host and varies with plant genotypes and the soil physicochemical environment (4–8). Whether and how the complex rhizosphere microbiome affects root metabolite composition and exudation remain unclear (9, 10). Determining the mechanisms that plants use to shape their microbiomes is thus an ongoing quest in the field of plant-microbe interactions. A molecular understanding of microbial responses elicited by specific metabolites and the impact of environmental variables on plant-microbe interactions is thus needed to establish the foundational knowledge required to capitalize on beneficial microbial traits that can be applied for the optimization of resilient and sustainable cropping systems.

### Status of current visualization technologies.

Due to the opaque nature and the complexity of the soil environment, capturing rhizosphere interactions in a meaningful ecosystem context is challenging (11). Traditionally, bacterial root colonization has been examined under aseptic laboratory conditions that are tightly controlled. Simplified synthetic bacterial communities and sophisticated microfluidics devices have advanced the study of plant-microbe and microbe-microbe interactions (12, 13). For example, with a microfluidics system for tracking root interactions, Massalha et al. (12) mapped microbial density changes over time and the community composition along the root. These systems take advantage of microscopy-based imaging of intact plant roots with their associated microbiomes. EcoFABs (fabricated microbial ecosystems) (14, 15) represent another concept for standardized and reproducible science that is customizable for plant-microbe interactions. This approach capitalizes on controlling all input variables and organisms that are studied within the EcoFABs. Genetically tractable, well-characterized model plants such as *Brachypodium*, *Setaria*, and *Arabidopsis* and model bacterial and fungal isolates are being used in EcoFABs that can then be customized to address specific scientific questions. A major downside to these approaches is that they do not mirror the true dynamics of the natural belowground environment in soil. Additional limitations pertain to the highly manipulated and constrained growing environments utilized by these systems, the minimal size of the units, and the short temporal scales used for experiments. Chemical imaging is another popular method for visualizing molecular-level details underlying plant-microbe interactions in the rhizosphere. For example, time of flight secondary ion mass spectrometry (ToF-SIMS) (16) and matrix-assisted laser desorption ionization (MALDI)–time of flight mass spectrometry combine spectral, spatial, and temporal information to generate a visual image of a given sample. Although these methods provide spatially resolved molecular information at a snapshot in time, elaborate sample processing techniques and extensive data acquisition and data processing times reduce the throughput of these mass spectrometry-based techniques. The destructive nature of the chemical imaging methods and sample size limitations make these approaches less attractive for studying whole root systems and the associated rhizosphere microenvironments. The majority of the above-stated techniques capture two-dimensional (2D) information, even though the interactions in native environments are not restricted to two dimensions. Stand-alone root imaging techniques ranging from scanning root systems on flatbed scanners to imaging using X-ray computed tomography (XCT), neutron tomography (NT), nuclear magnetic resonance (NMR) imaging, shovelomics, and photogrammetry are popular means of visualizing root systems (17, 18). Genetically manipulated root systems with fluorescent and bioluminescent reporters have also been used in investigating plant-microbe interactions (19). One advantage of noninvasive root imaging techniques is the possibility to record the same sample over time during growth and visualization of dynamic root development. However, due to limitations in resolution, it is not possible to capture details of the microenvironment and dynamic rhizosphere interactions using these stand-alone root imaging methods. Because imaging of plant-microbe interactions belowground remains challenging, there is still a need for robust alternatives for gaining a spatially resolved mechanistic understanding of this hidden half of the soil ecosystem. Image processing as well as three-dimensional (3D) modeling are equally important for the success of illustrating and extracting the 3D microenvironment of the rhizosphere. Thus, parallel efforts are under way to improve the computational and informatics support that goes hand in hand with new advances in rhizosphere imaging.

### New opportunities for 3D imaging of plant-microbe interactions in the rhizosphere.

**FIG 1:**
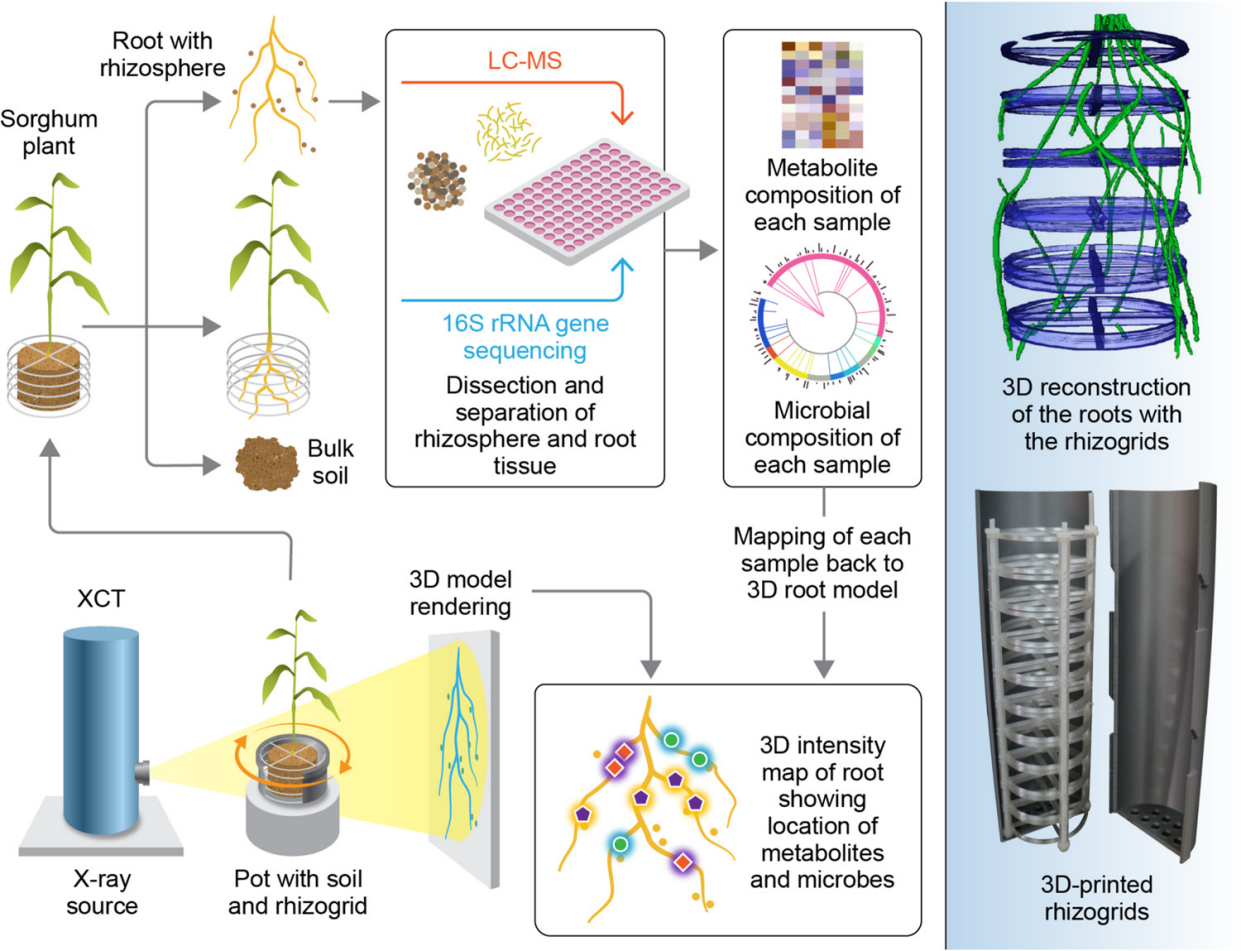
Three-dimensional root-rhizosphere cartography. In this example, sorghum was planted in soil using pots equipped with rhizogrids. A 3D image of the root was generated by X-ray computed tomography (XCT). Following 3D rendering of the root structure, the root and adhering soil were extracted from the pot. Loosely adhered soil was removed from the root while the rhizosphere remained attached. The root with its rhizosphere was segmented into 1-cm fragments, assigned coordinate positions, and separated into root and rhizosphere (root wash-off) fractions for metabolite and microbial profiling using liquid chromatography-mass spectrometry and 16S rRNA gene sequencing, respectively. Metabolite and microbial data were sequentially mapped back to the XCT-derived root image. (Figure created by Christer Jansson and Nathan Johnson [© Jansson and Johnson, reproduced with permission].)

**FIG 2:**
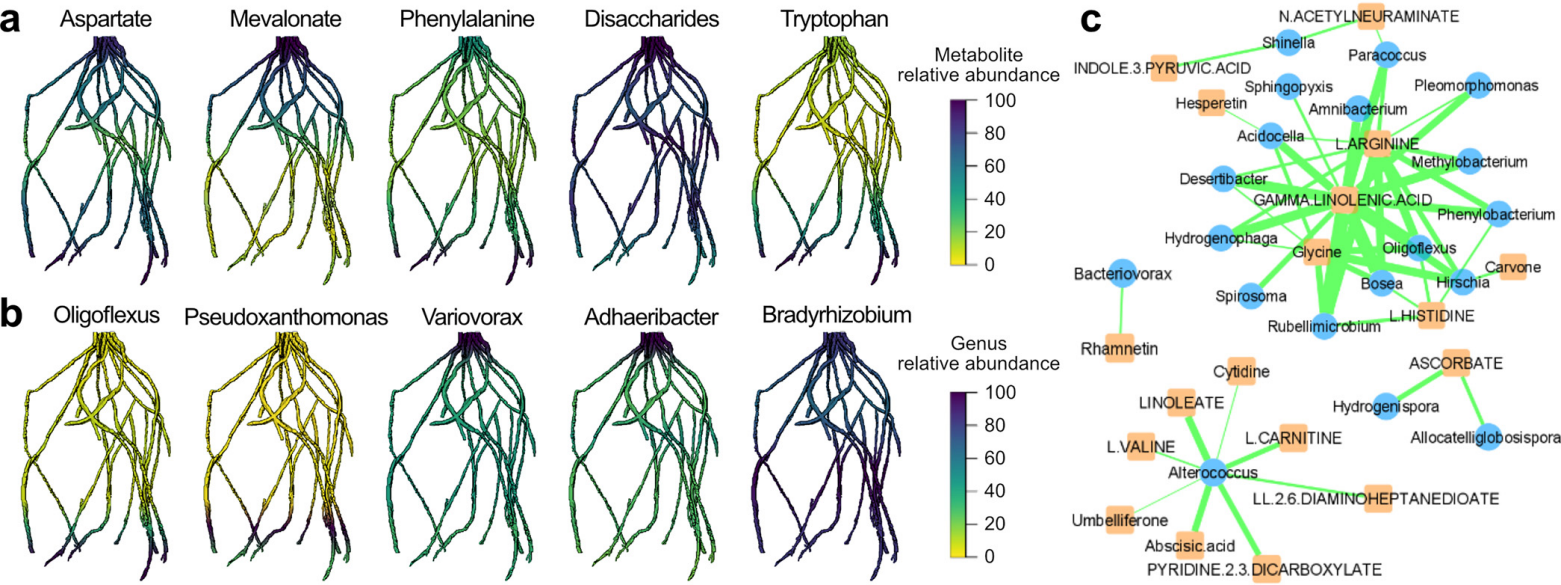
Spatially resolved metabolite abundance and microbial community composition along the sorghum root system illustrated as overall gradients along the rooting depths. (a and b) Five selected metabolites (a) and five selected bacterial genera (b) are visualized along the same root reconstruction. Using the rooting depth and relative abundance of each of the selected metabolites and microbial genera, the root system was manually colored to generate the root illustrations. (c) 16S rRNA gene amplicon and metabolomics data for different rooting depths were integrated to infer a network linking microbial genera and metabolites based on positive Pearson correlations. Microbial genera are indicated with blue circles, and metabolites are indicated with orange squares. The thickness of the green-line connections indicates the strength of the correlations between the linked metabolites and microbial genera.

One exciting new opportunity for 3D imaging of microbial interactions with roots is to capitalize on recent developments in the combined 3D mapping of microbes and metabolites as demonstrated for the Human Microbiome Project (20, 21). One challenge that we have with soils is their heterogeneity and opaqueness. Therefore, we need additional techniques to visualize and differentiate between the roots and the surrounding soil environment. In this commentary, we advocate for a newly developed 3D root cartography platform for capturing rhizosphere interactions in a soil environment, with root exudate profiles and microbial community membership mapped along the root system. As illustrated in Fig. 1, customizable 3D printed grids (rhizogrids) and pots are utilized for the growth experiments. The rhizogrids are fitted within the pots and act as scaffolds to offer physical support for the root system once removed from the pot. The rhizogrids also serve to provide 3D coordinates for reconstructing the 3D root image from excised root segments. For this proof-of-concept study, we used soil-grown sorghum [Sorghum bicolor (L.) Moench]. The intact root system was first imaged using XCT. Subsequently, root segments with rhizosphere soil attached were excised from each quadrant of the rhizogrid layers, from the bottom to the top of the pot. The rhizosphere soil was recovered by washing the roots. Each rhizosphere sample was then screened for its associated microbiome and metabolites. High-resolution liquid chromatography-coupled mass spectrometry (LC-MS) was used for metabolite profiling (22), and 16S rRNA amplicon sequencing (23) was used for microbial community composition and relative abundance measurements. Metabolite and microbial data were mapped back to the root system to visualize the occurrence of selected metabolites and microbial genera along the root length. Correlative networking approaches (24) were used to infer linkages between specific metabolites and microbial genera (Fig. 2).

Our 3D root cartography platform enabled the testing of plant-microbe interactions in the rhizosphere at a relatively large scale (up to ∼500 mm) and in a setting that includes natural soil-level interactions occurring at the root-rhizosphere interface. Moreover, the rhizogrids can be customized to accommodate various root systems of interest as well as different growth substrates such as sand, vermiculite, and soils collected from agricultural fields and marginal lands. It is important to note that the growth medium should be tested to ensure enough contrast in XCT scans between the root system and the selected growth substrate to generate a robust 3D reconstruction of the root system architecture.

### Conclusions and future directions.

The presented example of 3D root cartography demonstrates the potential for utilizing this approach in visualizing spatially resolved molecular information on plant-microbe interactions belowground. These molecular interactions include specific metabolic interactions between microbes and root-exuded metabolites as well as colonization by and recruitment of specific microorganisms along the root. The metabolic versatility of the rhizosphere microbiome and the positive recruitment of specific microbial taxa can then be interpreted using correlation networks. The interactions themselves can be inferred from the various molecular data generated within the 3D root cartography platform to help visualize the specific interactions and the rhizosphere plant-microbe interactomes. This new approach has the potential to be extended to visualize how the native soil microbiome responds to abiotic perturbations such as drought and nutrient deficiencies and biotic stresses imposed by pathogens or the introduction of genetically engineered or other nonnative microbial strains. This approach can also be used to assess how specific members of the rhizosphere microbiome are recruited at different locations on the root by challenging the same root system with various native and synthetic microbiomes. We note that 3D visualization of microbial recruitment, colonization, and competition can be further enhanced by utilizing fluorescently labeled microbial strains coupled with cell sorting. The rhizogrid-based 3D root cartography platform can be expanded to include protein and transcript profiling. For example, targeted proteomics and metabolomics combined with quantitative transcript profiling of excised and barcoded root segments will inform on active metabolic pathways in different parts of the 3D root space and under different environmental settings, such as with or without associated microbes, and under drought or nondrought conditions. Complementary enzyme assays of the root segments will shed further light on metabolic processes. We also anticipate that direct 3D mapping of the chemical and physical properties of soil will become technically feasible and available within a 5-year time span. Coupling the properties of the surrounding soil to results from root and rhizosphere analyses will be necessary for a comprehensive understanding of biological processes in a plant-microbe-soil continuum. Much progress has been made in 2D soil investigations using a variety of optodes, and it is reasonable to assume that major inroads in 3D soil mapping will be made in the near future. Meanwhile, soil can be collected from rhizogrid coordinates and subjected to a multitude of approaches, such as near-edge X-ray spectromicroscopy, synchrotron X-ray absorption spectroscopy, and synchrotron-based microfluorescence spectroscopy (25). Universal sample holders can be developed for correlative analyses of soil in very much the same vein as we have demonstrated for surface imaging of root samples from the model plant Brachypodium distachyon (16). The 3D root cartography platform can also be aligned with stable isotope probing (SIP). As an example, CO_2_ labeling with ^13^C can be employed in order to monitor the partitioning of recent photosynthates within the 3D root system and how this partitioning is affected by microbes and environmental perturbations. Our research with SIP has revealed that plants rapidly shift carbon allocation between aboveground versus belowground biomass and partitioning into different metabolites, including root exudates, in response to drought stress or in the presence versus the absence of microbes. Embedding this research to 3D root imaging will allow us to spatially resolve such shifts in the 3D root space. We also posit that the 3D root cartography platform that we present here offers a compelling opportunity to capitalize on recent developments in both sequencing technologies that enable the characterization of complex microbiomes with high throughput and advances in metabolomic approaches that allow the functional characterization of microbial pathways and the metabolic interactions of microbial communities with plants as they respond to diverse environmental conditions. To better visualize spatially resolved information and identify molecular and microbial hot spots along the 3D reconstructed root systems, we are currently developing a Python-based software package for data-type-agnostic visualization of features in XCT-derived 3D images.

The metabolome of an organism is closest to its final phenotype, is the first to respond to the surrounding environmental changes, and therefore plays an important role in defining the final phenotype (22). Rhizosphere microbiomes can be augmented or selected for beneficial microbial traits that play a key role in plant productivity and fitness. We anticipate that obtaining an accurate assessment of the relationship between the plant exudate metabolome and microbial community structure in the rhizosphere environment can significantly impact plant breeding and plant genetic engineering research, thus redefining future agricultural practices.
